# Fabrication of hierarchically porous TiO_2_ nanofibers by microemulsion electrospinning and their application as anode material for lithium-ion batteries

**DOI:** 10.3762/bjnano.8.131

**Published:** 2017-06-22

**Authors:** Jin Zhang, Yibing Cai, Xuebin Hou, Xiaofei Song, Pengfei Lv, Huimin Zhou, Qufu Wei

**Affiliations:** 1 Key Laboratory of Eco-textiles, Ministry of Education, Jiangnan University, Wuxi, Jiangsu 214122, People’s Republic of China; 2 College of Textile and Clothing, Jiangnan University, Wuxi, Jiangsu 214122, People’s Republic of China

**Keywords:** anode, hierarchically porous TiO_2_ nanofibers, lithium-ion batteries, microemulsion electrospinning (ME-ES), multichannel

## Abstract

Titanium dioxide (TiO_2_) nanofibers have been widely applied in various fields including photocatalysis, energy storage and solar cells due to the advantages of low cost, high abundance and nontoxicity. However, the low conductivity of ions and bulk electrons hinder its rapid development in lithium-ion batteries (LIB). In order to improve the electrochemical performances of TiO_2_ nanomaterials as anode for LIB, hierarchically porous TiO_2_ nanofibers with different tetrabutyl titanate (TBT)/paraffin oil ratios were prepared as anode for LIB via a versatile single-nozzle microemulsion electrospinning (ME-ES) method followed by calcining. The experimental results indicated that TiO_2_ nanofibers with the higher TBT/paraffin oil ratio demonstrated more axially aligned channels and a larger specific surface area. Furthermore, they presented superior lithium-ion storage properties in terms of specific capacity, rate capability and cycling performance compared with solid TiO_2_ nanofibers for LIB. The initial discharge and charge capacity of porous TiO_2_ nanofibers with a TBT/paraffin oil ratio of 2.25 reached up to 634.72 and 390.42 mAh·g^−1^, thus resulting in a coulombic efficiency of 61.51%; and the discharge capacity maintained 264.56 mAh·g^−1^ after 100 cycles, which was much higher than that of solid TiO_2_ nanofibers. TiO_2_ nanofibers with TBT/paraffin oil ratio of 2.25 still obtained a high reversible capacity of 204.53 mAh·g^−1^ when current density returned back to 40 mA·g^−1^ after 60 cycles at increasing stepwise current density from 40 mA·g^−1^ to 800 mA·g^−1^. Herein, hierarchically porous TiO_2_ nanofibers have the potential to be applied as anode for lithium-ion batteries in practical applications.

## Introduction

The increasing demand for energy has heightened the need for the development of renewable resources because fossil fuels, which are currently the most common energy resources, are a finite resource [1–4]. Battery industry, which is considered to be a vital part in the field of renewable resources, has attracted much attention in recent years. Substantial effort has been made to improve the performance of electrode materials for lithium-ion batteries aiming at aspects including safety, energy density, lifetime and power density [5–8].

So far, among all the commercial lithium-ion batteries, graphite plays an extremely important role in anode materials; nevertheless, structural deformation, electrical disconnection and the initial loss of capacity hinder its further development [9–10]. Titanium dioxide (TiO_2_) is considered to be an alternative anode material to graphite, which can be attributed to the superior advantages of titanium dioxide such as low-cost, eco-friendliness, nontoxicity and high abundance [10]. Furthermore, safety and stability of titanium dioxide are higher than those of graphite, because since Li ions can be inserted into the TiO_2_ matrix at higher voltages (at least 1.5 V vs Li/Li^+^) [11–13]. TiO_2_ possesses a high theoretical specific capacity of 335 mAh·g^−1^ [14–16]. However, the poor rate performance and cycling performance has hindered the practical application of titanium dioxide in lithium-ion batteries. The low conductivities of ions and bulk electrons as well as the aggregation of nanoparticles are the major causes of poor rate performance and cycling performance.

One effective method to improve the electrochemical performance of TiO_2_ is to prepare nanostructures [17–20] that have advantages over normal structures including a large specific surface area, a high electrolyte–electrode contact area and excellent mass transport of products or reactants to active sites inside meso- or micropores. One-dimensional (1D) nanostructures such as nanofibers, nanotubes, nanowires and nanorods are considered to be promising electrode materials for excellent lithium-ion battery performance. This is partly because of their small size enabling fast electron transport and decreasing retardation at the interface [21–22]. Electrospinning, a simple and versatile method, has been widely used in fabricating various nanostructures. The structure of nanofibers can be conveniently adjusted by changing the experimental parameters, the composition of the precursor solution and the structure of the spinneret [23–25].

Core–shell nanofibers are normally prepared by coaxial electrospinning. However, coaxial electrospinning limits the further development of core–shell nanofibers because of its complicated spinneret, the limited number of suitable fluids and the unstable structure of the fibers. Recently the method of ME-ES has been developed to fabricate hierarchical nanofibers without the need of a complex spinneret. It is a simple and versatile way to prepare nanofibers with hollow or multichannel structures [26]. More importantly, various inorganic nanofibers can be fabricated easily by ME-ES including TiO_2_, SiO_2,_ ZrO_2_, SnO_2_, V_2_O_5_, GeO_2_ and Al_2_O_3_. A microemulsion system was composed of a metal alkoxide and an oil phase as, respectively, continuous phase and dispersion phase in the system [27]. The microemulsion solution was electrospun to form nanofibers and, after calcination, the as-spun nanofibers partly decomposed to hierarchically porous inorganic nanofibers [26]. Chen et al. reported the fabrication of hierarchical TiO_2_ nanorods via ME-ES and the application as photoanode material for dye-sensitized solar cells [25]. According to Shi et al., highly porous SnO_2_/TiO_2_ composite nanofibers were prepared successfully by ME-ES and subsequent calcination [28].

There are various reports about porous TiO_2_ nanofibers as anode for LIB. Cho et al. prepared fiber-in-tube TiO_2_ nanofibers as anode material for LIB and the initial discharge and charge capacity were 231 and 170 mAh·g^−1^, respectively [29]. Furthermore multichannel hollow TiO_2_ nanofibers were fabricated by Tang et al. and demonstrated excellent rate capability and cycling performances when used as anode in LIB [30]. However, there is no report about hierarchically porous TiO_2_ nanofibers fabricated via ME-ES used as anode material for LIB. So in this work, the ME-ES solution was firstly electrospun to prepare nanofibers, then as-spun nanofibers were calcined at 500 °C to form porous TiO_2_ nanofibers. The electrochemical performances of hierarchically porous TiO_2_ nanofibers as anode material for LIB were investigated in detail.

## Experimental

### Materials

Tetrabutyl titanate (TBT), paraffin oil, absolute ethanol, acetic acid and cetyltrimethylammonium bromide (CTAB) were supplied by Shanghai Chemical Regents Co. (Shanghai, China). Additionally, polyvinyl pyrrolidone (PVP) (*M*_w_ = 1300000) was purchased from Tianjin Bodi Chemical Reagent Co., Ltd. (Tianjin, China). All chemicals were used as received without further purification.

### Preparation of the spinning solution

0.5 g PVP and 0.3 g CTAB were firstly dissolved into 6.5 g ethanol and 0.4 g acetic acid followed by vigorously stirring to form a homogeneous solution. Subsequently, 1 g paraffin oil and stoichiometric amounts of TBT with different TBT/paraffin oil ratios (w/w) of 2.25, 1.9 and 1.55 were added slowly under stirring for 12 h to obtain a microemulsion.

### Preparation of porous TiO_2_ nanofibers

The prepared microemulsion was filled in a 30 mL syringe with a blunt-end stainless steel needle, which had an inside diameter of 0.3 mm. Then the microemulsion was electrospun into nanofibers by a custom-made electrospinning setup consisting of a high voltage power supply, a syringe pump and a grounded nanofiber collector covered with aluminium foil. During electrospinning, a positive voltage of 20 kV was applied between the needle tip and grounded collector at a distance of 15 cm. The flow rate of the spinning solution was maintained at 2 mL·h^−1^. The as-spun nanofibers with TBT/paraffin oil ratios of 2.25, 1.9 and 1.55 were marked as sample A_1_, sample B_1_ and sample C_1_, respectively.

Subsequently, the as-spun nanofibrous mats were calcined in a quartz tube furnace in air atmosphere at 500 °C for 2 h to yield hierarchically porous TiO_2_ nanofibers. The porous TiO_2_ nanofibers obtained after calcination with TBT/paraffin oil ratios of 2.25, 1.9 and 1.55 were named as sample A_2_, sample B_2_ and sample C_2_, respectively. In other words, sample A_2_ corresponded to the calcined sample A_1_, sample B_2_ and sample C_2_ were the same naming method as sample A_2_.

### Characterization

Thermogravimetric analysis (1100SF) was employed to characterize the thermal decomposition of as-spun TiO_2_ nanofibers in air atmosphere with a heating rate of 10 °C/min. X-ray diffraction patterns of porous TiO_2_ nanofibers were recorded on a Bruker D8 Advance X-ray diffractometer with Cu Kα radiation (wavelength λ = 1.54 Å) at a scanning speed of 4 °C/min. A Hitachi field emission scanning electron microscope (FE-SEM, SU4800) was employed to observe the surface morphology of porous TiO_2_ nanofibers. Prior to the FE-SEM examination, all the specimens were sputter-coated with gold to avoid charge accumulations. Transmission electron microscopy was conducted on a JEOL JEM-2100 transmission electron microscopy unit at an accelerating voltage of 120 kV. The specific surface area and pore structure of porous TiO_2_ nanofibers were characterized with a physisorption analyzer (ASAP 2020, Micromeritics).

### Electrochemical measurements

The electrodes were prepared by mixing thoroughly porous TiO_2_ nanofibers (60 wt %), carbon black (20 wt %) and poly(tetrafluoroethylene) (20 wt %) to form a homogeneous paste. Subsequently, the paste was coated uniformly on the nickel net and then dried in a vacuum oven at 60 °C for 12 h. Prior to cell assembly, the electrodes were cut into circular disks with a diameter of 16 mm. The testing cells employed Celgard 2300 membrane as separator, lithium foil as both reference and counter electrode, and 1 M LiPF_6_ dissolved in ethylene carbonate (EC), dimethyl carbonate (DMC) and ethylene methyl carbonate (EMC) (1:1:1, v/v/v) as the electrolyte, respectively. Coin cells were assembled in an argon-filled glove box. The galvanostatic discharge–charge tests were conducted at the constant current density of 40 mA·g^−1^ in the voltage range of 0.01–3.0 V (vs Li^+^/Li). The cyclic voltammetry curves were tested at a scanning rate of 0.1 mV/s over the voltage range of 0.01–3.0 V (vs Li^+^/Li) with a CHI660e electrochemical workstation.

## Results and Discussion

### Thermal properties and structural morphology of porous TiO_2_ nanofibers

**Figure 1:**
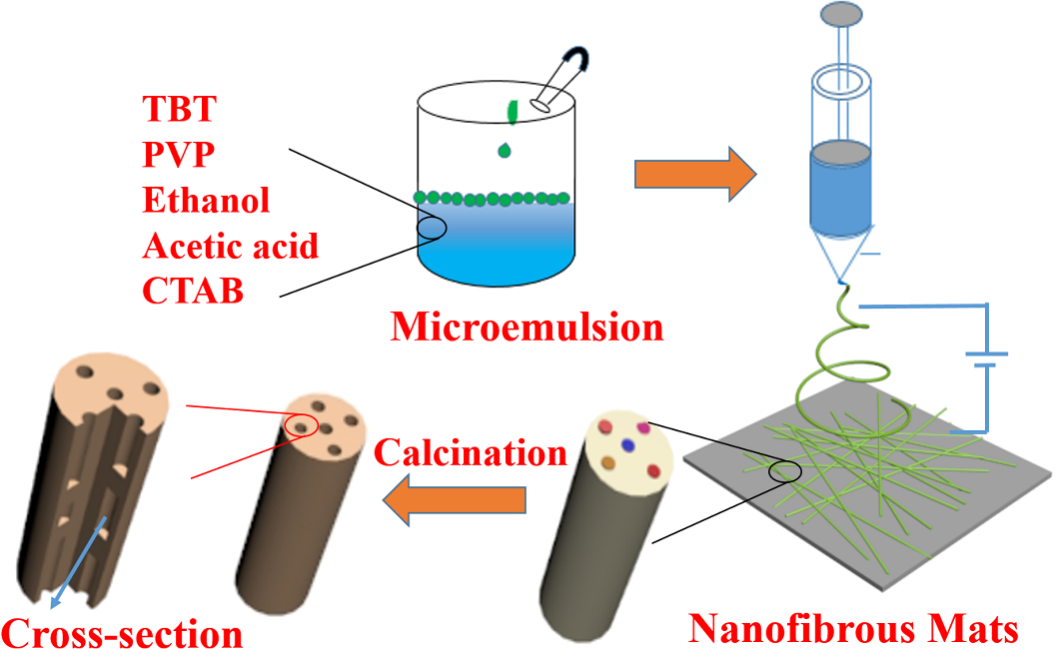
Fabrication procedure of porous TiO_2_ nanofibers via ME-ES.

The fabrication process of hierarchically porous TiO_2_ nanofibers is presented in Figure 1. When paraffin oil was added dropwise into the solution, it floated on the surface of the solution. After stirring vigorously, the opaque precursor solution was prepared successfully. During the process of electrospinning, the viscous liquid was elongated quickly accompanied by the evaporation of solvent as well as the hydrolysis condensation of TBT. Herein, despite the fact that the oil droplets in the microemulsion were stable with a diameter of about 20 nm, several small oil droplets merged into larger ones and then were stretched strongly by electrostatic force along the direction of fluid jet, thus forming oriented and axially aligned oil droplets. After calcination, the axially aligned oil droplets would be removed, resulting in axially aligned pores with the diameters of tens of nanometers. It meant that the axially aligned pores were the vacancies of paraffin oil.

**Figure 2:**
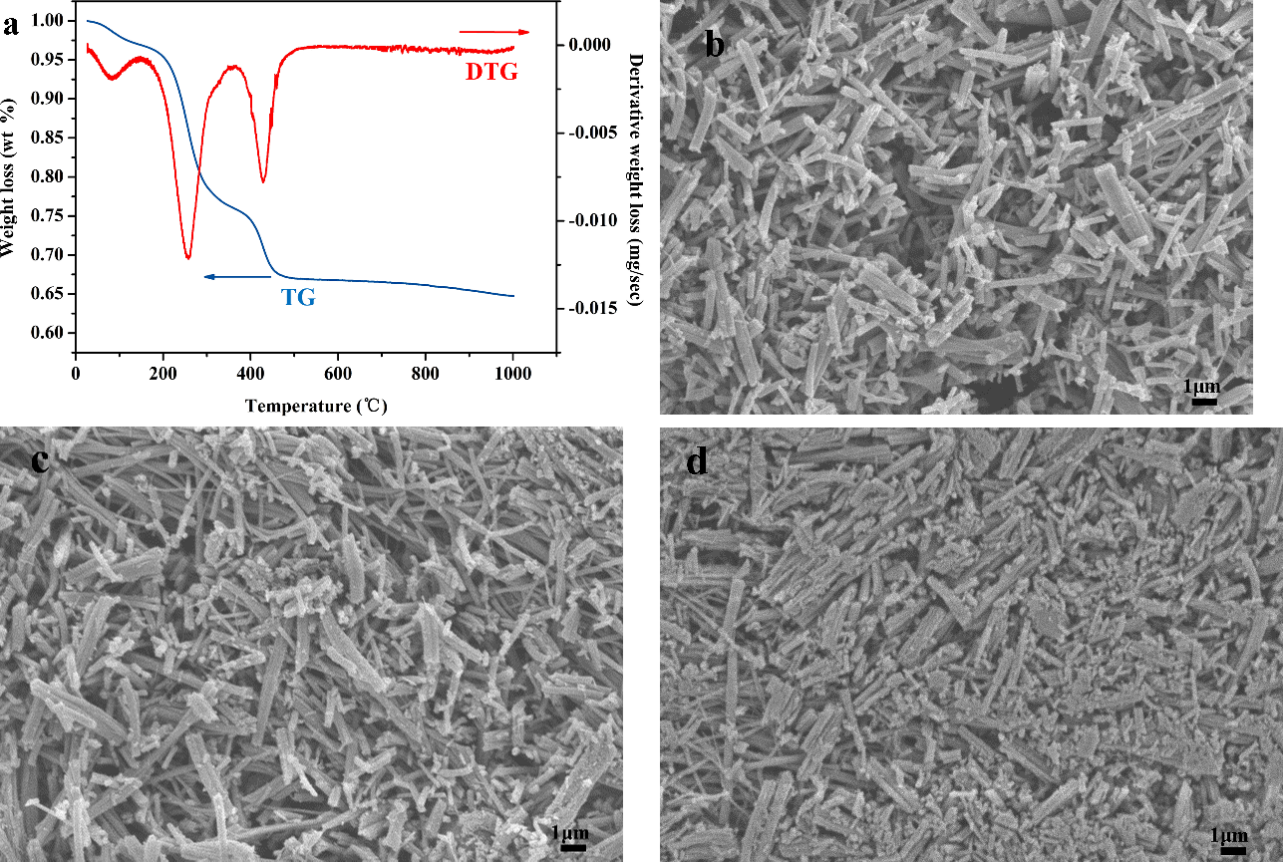
(a) TG and DTG curves of as-spun TiO_2_ nanofibers; surface SEM images after calcination of sample A_2_ (b), sample B_2_ (c) and sample C_2_ (d).

Thermogravimetry (TG) and differential thermogravimetry (DTG) curves of as-spun sample A_1_ are shown in Figure 2a. It can be seen that the total weight loss was approximately 35%, and whole decomposition process could be divided into three steps. The first step occurred between 25 to 150 °C and can be attributed to the evaporation of residual solvent and water. The second step from 160 to 370 °C corresponds to the major decomposition process of long molecular chains of PVP and paraffin oil as well as to the partial decomposition of TBT molecules, resulting in weight loss of 20%. The final decomposition step occurred in the temperature range from 380 to 500 °C and corresponds to the further decomposition of molecular chains of PVP and paraffin oil as well as the complete degradation of TBT, causing a weight loss of approximately 8%. It should be noted from Figure 2 that there was no obvious weight loss above 500 °C, suggesting the formation of stable TiO_2_ nanofibers. Figure 2b–d displays the surface SEM images of sample A_2_, sample B_2_ and sample C_2_. It can be observed that after calcination, the nanofibers had the length of a few micrometers and a diameter of hundreds of nanometers. Moreover, from Figure 2b–d, the nanofibers became increasingly brittle due to the decreased content of TBT in the microemulsion system.

**Figure 3:**
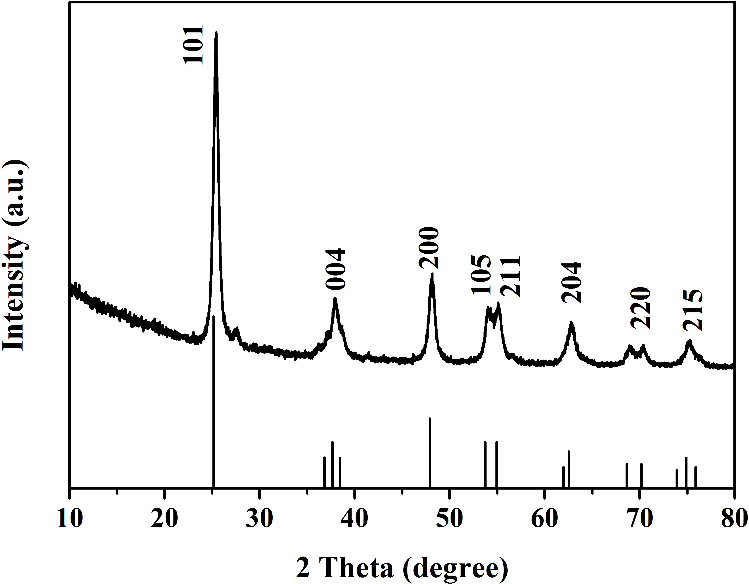
Representative XRD pattern of porous TiO_2_ nanofibers.

Figure 3 shows the representative XRD pattern of sample A_2_ calcined at 500 °C for 2 h in air atmosphere. All the peaks can be indexed to anatase TiO_2_ (JCPDS, No. 21-1272). The diffraction peaks at 2θ = 25.281, 37.800, 48.049, 53.890, 55.060, 62.688, 70.309 and 75.029° correspond to the (101), (004), (200), (105), (211), (204), (220) and (215) reflections, respectively. It is notable that there was no peak of any other phase in Figure 3, thus confirming the pure phase of prepared porous TiO_2_ nanofibers. Considering that all the porous TiO_2_ nanofibers were calcined at 500 °C, all of them should be expected to be anatase TiO_2_. More importantly, among the various polymorphs of TiO_2_, the anatase polymorph possesses an open crystal structure resulting from the stacking of zigzag units, which would benefit the intercalation/deintercalation of Li^+^ ions.

**Figure 4:**
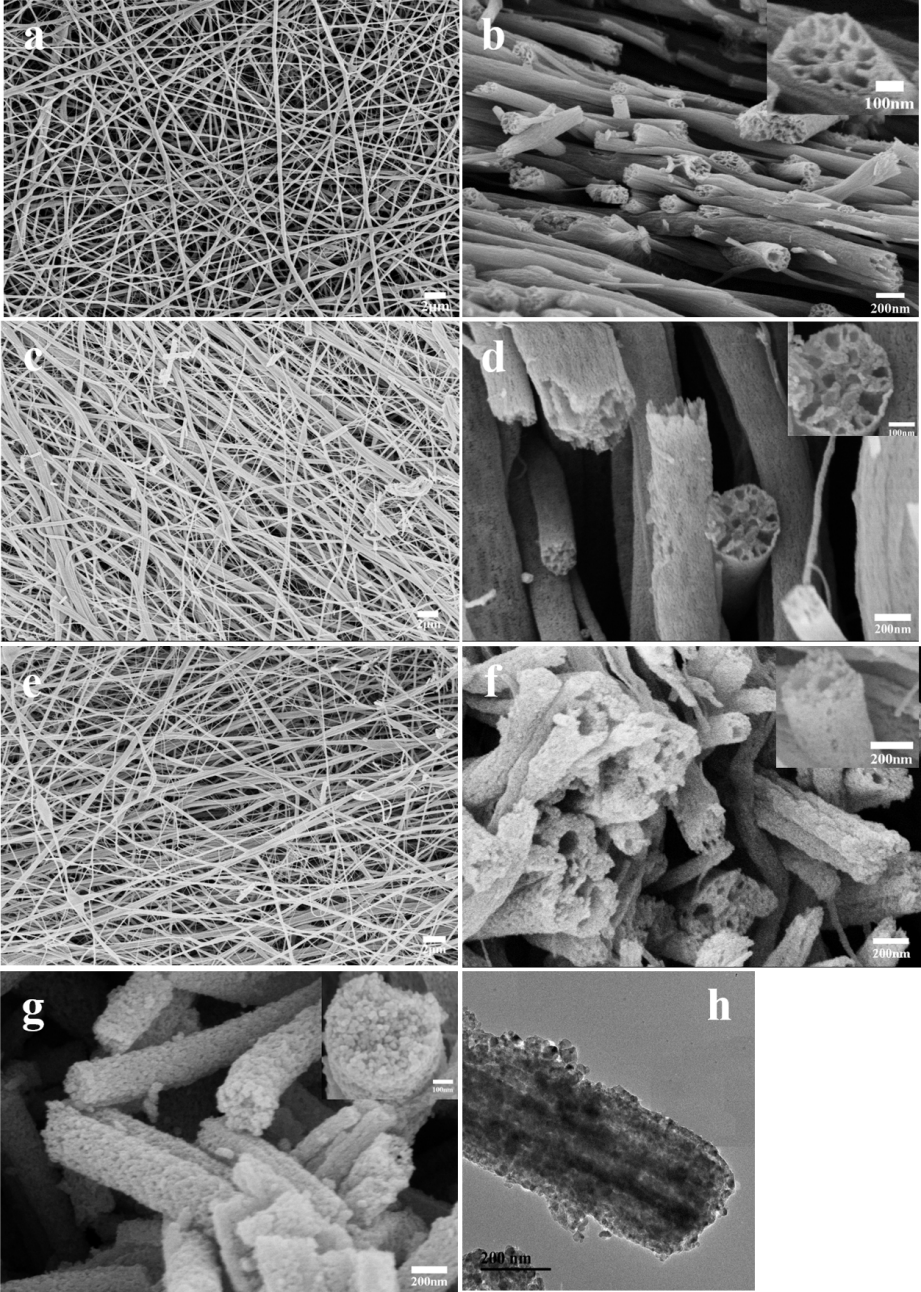
Surface SEM images of sample A_1_ (a), sample B_1_ (c), and sample C_1_ (e); cross-sectional SEM images of sample A_2_ (b), sample B_2_ (d), sample C_2_ (f) and solid TiO_2_ nanofibers (g), inset SEM images were the corresponding images with higher magnification; and representative TEM images of sample A_2_ (h).

Figure 4 displays the surface SEM images of sample A_1_ (a), sample B1 (c), and sample C_1_ (e) as well as cross-sectional SEM images of sample A_2_ (b), sample B_2_ (d), sample C_2_ (f) and solid TiO_2_ nanofibers (g), and representative TEM images of sample A_2_. In addition, the inset SEM images are the corresponding images with the higher magnification. The as-spun nanofibers have a smooth surface and relatively uniform distribution. Nevertheless, it can be observed that with decreasing relative content of TBT/paraffin oil, the surface morphology looks worse, and the distribution of the fiber diameter becomes increasingly non-uniform. This is because the butoxyl groups in TBT serve as additional surfactant, which is advantageous to form the stable interfaces between the two phases, thus forming a stable microemulsion. In other words, the decreased content of TBT caused increasing instability of the microemulsion, so that the morphology of the nanofibers became worse. In addition, the most significant phenomenon is that the number of channels in each nanofiber decreased as the content of paraffin oil increased. This is because the pores stemmed from the vacancies of oil droplets after calcination, and a larger oil droplets were formed when more paraffin oil was added into the microemulsion [31]. The SEM image of unmodified TiO_2_ nanofibers in Figure 4g reveals solid structures without pores in the cross-section. This sample was used as reference in the following investigation. As shown in Figure 4h, a representative TEM image of a single porous nanofiber confirms that the pores inside the fibers were oriented and axially aligned, which may be caused by both the strong stretching force from the strong electric field and the formation of larger oil droplets during electrospinning [25].

**Figure 5:**
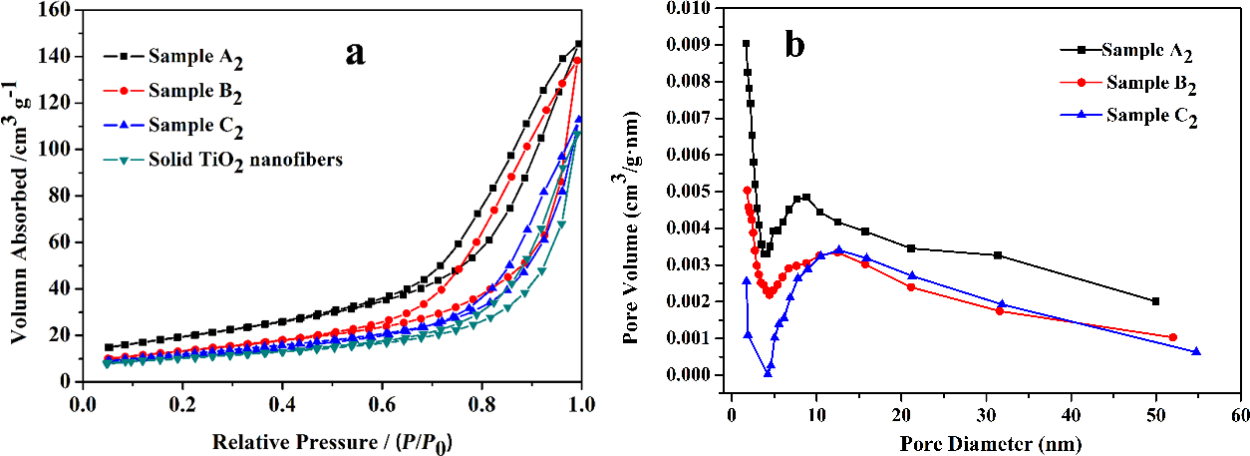
(a) Nitrogen adsorption–desorption curves of sample A_2_, sample B_2_, sample C_2_ and solid TiO_2_ nanofibers; (b) pore-size distribution of sample A_2_, sample B_2_, sample C_2_.

Nitrogen adsorption–desorption isotherms of sample A_2_, sample B_2_, sample C_2_ and solid TiO_2_ nanofibers are shown in Figure 5. All isotherms in Figure 5 belong to type IV accompanied with a hysteresis loop located at higher relative pressures [32–33]. The specific surface areas of sample A_2_, sample B_2_, sample C_2_ and solid TiO_2_ nanofibers were approximately 69.9, 48.2, 43.1 and 35.6 m^2^/g, respectively. It is noteworthy that the specific surface area of sample A_2_ was almost two times that of solid TiO_2_ nanofibers, which demonstrated the fact that ME-ES was indeed an effective method to enlarge the specific surface area. The specific surface area plays a very important role in the performance of electrodes for lithium-ion batteries. The merits of porous nanofibers with a higher specific surface area lie in the higher lithium-ion flux across the interfaces and the larger contact area between the electrode and electrolyte [2,34–35]. Herein, sample A_2_ should have the best performances as the electrode of lithium-ion battery in theory. In addition, Figure 5b displays the pore-size distribution of sample A_2_, sample B_2_ and sample C_2_. According to the analysis of pore-size distribution with the Barrett–Joyner–Halenda (BJH) method, the average pore size increased gradually, and the average pore diameter was 17.8, 18.5 and 20.1 nm for sample A_2_, sample B_2_, sample C_2_, respectively. Moreover, it can be observed clearly that the average diameter of macropores increased with the ratio of TBT/paraffin oil decreasing. The results confirmed the presence of mesopores and macropores in the porous TiO_2_ nanofibers.

### Electrochemical performances

The electrochemical properties of TiO_2_ nanofiber electrodes were investigated by cyclic voltammetry (CV) test using lithium foil as counter electrode and reference electrode. The CV curves of sample A_2_ and solid TiO_2_ nanofibers examined in the range of 0.01–3 V (vs Li^+^/Li) at a scanning rate of 0.1 mV/s are shown in Figure S1 (Supporting Information File 1). Sample A_2_ has obvious reduction and oxidation peaks at about 1.49 and 2.33 V. The reduction peak corresponded to the intercalation of Li^+^ into interstitial octahedral sites of anatase TiO_2_ via a phase transition from tetragonal anatase to orthorhombic Li_0.5_TiO_2_ according to the scheme: *x*Li^+^ + TiO_2_+ *xe*^−^ ↔ Li*_x_*TiO_2_ [36–37]_._ There is no big difference between sample A_2_ and solid TiO_2_ nanofibers (Figure S1, Supporting Information File 1).

**Figure 6:**
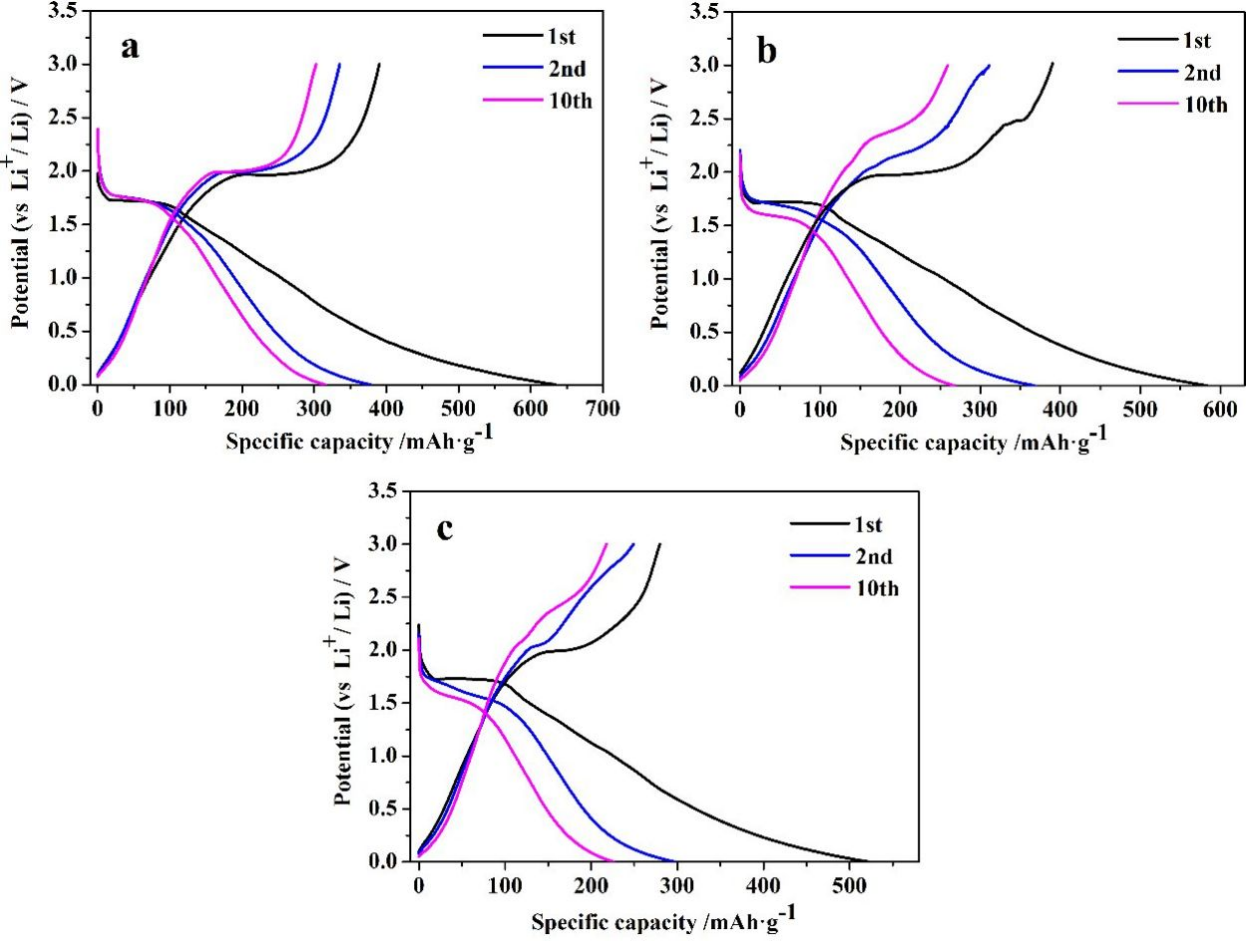
Galvanostatic charge–discharge curves of sample A_2_ (a), sample B_2_ (b) and sample C_2_ (c) for the first ten cycles at the current density of 40 mA·g^−1^.

Figure 6 shows the representative galvanostatic charge–discharge curves of sample A_2_ (a), sample B_2_ (b) and sample C_2_ (c) at a current density of 40 mA·g^−1^. It is apparent that all the charge or discharge profiles were similar, thus implying that the processes of lithium insertion or extraction were the same. There were obvious voltage plateaus in all discharge curves at around 1.72 V and charge curves at around 1.93 V, which were due to the phase transition between tetragonal anatase and orthorhombic Li_0.5_TiO_2_ [38]. The initial discharge capacities of sample A_2_, sample B_2_ and sample C_2_ were 634.72, 583.44 and 522.65 mAh·g^−1^, and the corresponding charge capacities were 390.42, 367.85 and 279.82 mAh·g^−1^, resulting in coulombic efficiencies of 61.51%, 63.05% and 53.54%, respectively. It was worth noting that the specific capacities increased accordingly as the specific surface area of the samples increased, which further confirmed that specific surface area plays a vital role in the performance of the LIB anode material. Sample A_2_ with a specific surface area of 69.9 m^2^·g^−1^ exhibited the best performance among all the samples, and was chosen for further investigation.

**Figure 7:**
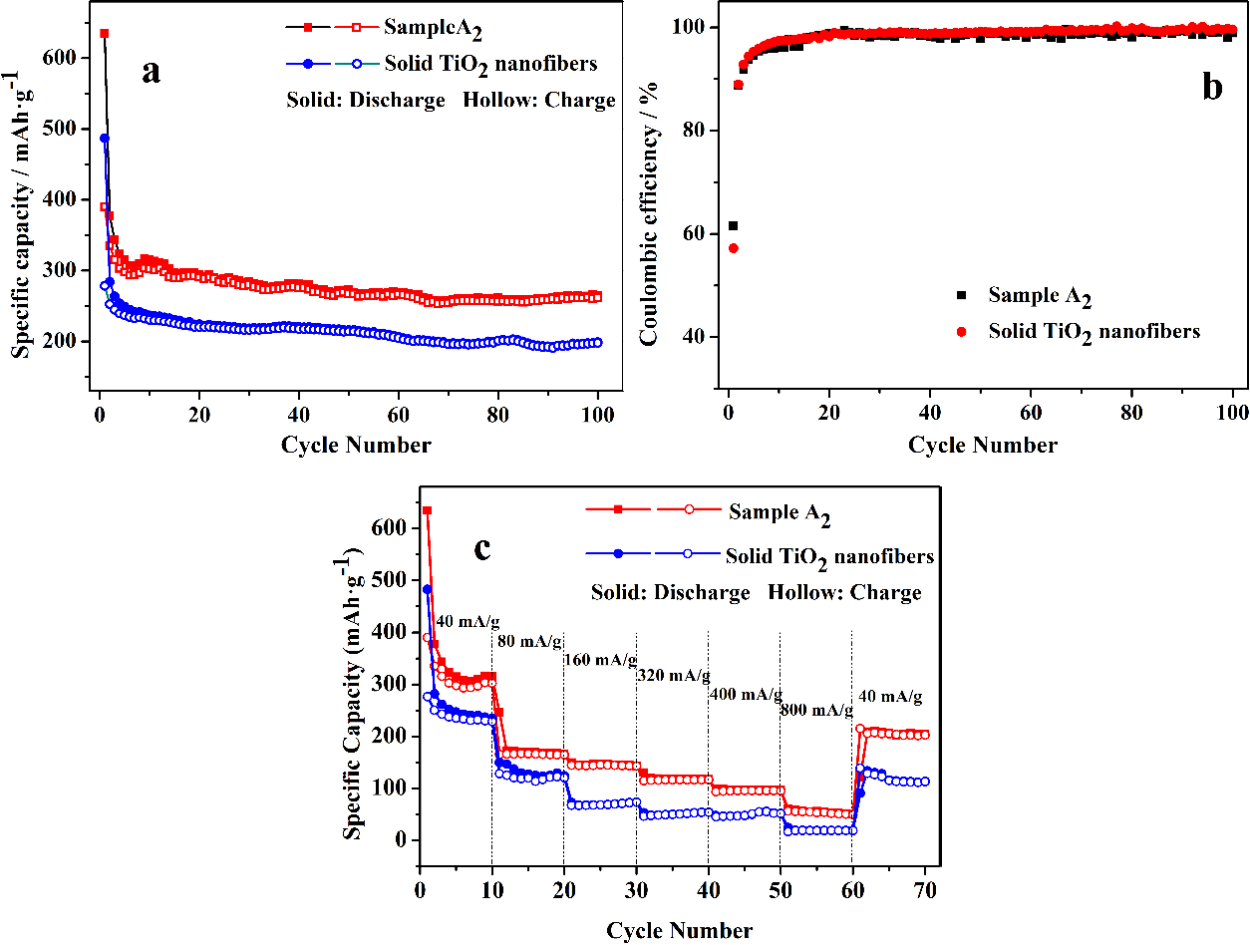
Comparison of cycling performance (a), coulombic efficiency (b) and rate capability (c) of sample A_2_ and solid TiO_2_ nanofibers.

Figure 7 provides the cycling performance (a), coulombic efficiency (b) and rate capability (c) of sample A_2_ and solid TiO_2_ nanofibers. As can be seen from Figure 7a, there was relatively large capacity fading in the first several cycles for both samples, which may be ascribed to the partial irreversible decomposition of the electrolyte, the incomplete decomposition of the solid–electrolyte interface (SEI) or a partial irreversible intercalation of Li^+^ into TiO_2_ nanofibers [30]. The capacity remains relatively stable in the following cycles. The 100th discharge (charge) capacity of sample A_2_ and solid TiO_2_ nanofibers were 264.56 (261.61) and 198.96 (198.12) mAh·g^−1^, respectively. It is apparent that the cycling performance of sample A_2_ shows significant improvement compared with solid TiO_2_ nanofibers, which can be attributed to the larger specific surface area of sample A_2_. As mentioned in Figure 7, the coulombic efficiency of sample A_2_ was 61.51% for the first cycle, whereas the coulombic efficiency of solid TiO_2_ nanofibers was 57.21%. And the coulombic efficiency remained relatively constant in the following cycles due to the relatively stable cycling performance. The rate capability plays a crucial role in the high-power applications of LIB, therefore rate capability of both samples was evaluated by stepwise increasing the current density. The specific capacity decreased gradually with increasing current density. The electrode delivered reversible discharge capacities of 315.14, 166.47, 142.55, 117.56, 96.42, 50.195 mAh·g^−1^ at the current density of 40, 80, 160, 320, 400, 800 mA·g^−1^, respectively. Despite the fact that the capacity could not recovered fully, the corresponding specific capacity of sample A_2_ was as high as 204.53 mAh·g^−1^ when the current density returned back to 40 mA·g^−1^, which was much higher than the values of solid TiO_2_ nanofibers. Sample A_2_ as anode for LIB demonstrated excellent rate capability, which stemmed mostly from the hierarchically porous structure and the large specific surface area.

Electrochemical impedance spectroscopy (EIS) was carried out on the electrode of sample A_2_ and solid TiO_2_ nanofibers. Nyquist impedance plots obtained for sample A_2_ and solid TiO_2_ nanofibers in a fresh cell are presented in Figure S2 (Supporting Information File 1). The plots for the two samples were similar, consisting of a compressed semicircle and a straight line. The semicircles in the high-frequency range represents the charge-transfer resistance (*R*_ct_) and the straight line in the low-frequency region represents the diffusion and accumulation process of lithium ions in the electrode [1,29,39]. As can be seen from Figure S2 (Supporting Information File 1), the charge–transfer resistance of sample A_2_ was about 235 Ω, whereas the charge–transfer resistance of solid TiO_2_ nanofibers was about 425 Ω. Herein, the lower charge–transfer resistance of sample A_2_ can be ascribed to the hierarchically porous structures of sample A_2_ facilitating charge transfer at the electrolyte–electrode interfaces [40–42] (Figure S2, Supporting Information File 1). Table 1 compares the electrochemical performances of sample A_2_ with other reference results.

**Table 1:** Comparison of electrochemical performances of different TiO_2_ nanostructures.

number	structures	reversible capacity (mAh·g^−1^)	charge–discharge rates (mA·g^−1^)	reference

1	TiO_2_ nanofibers with fiber-in-tube structure	170	200	[29]
2	mesoporous TiO2 nanotubes	108	335	[43]
3	multi-channel hollow TiO_2_ nanofibers	212	168	[30]
4	TiO_2_ nanoflakes	261	33	[44]
5	TiO_2_ hollow spheres	148	850	[45]
6	hierarchically porous TiO_2_ nanofibers	205	40	this work

## Conclusion

Hierarchically porous TiO_2_ nanofibers were fabricated successfully via single-nozzle microemulsion electrospinning. Moreover, multichannel TiO_2_ nanofibers displayed excellent lithium-ion storage properties compared with normal solid TiO_2_ nanofibers when used as anode for LIB. The initial discharge and charge capacities of porous TiO_2_ nanofibers with a TBT/paraffin oil ratio of 2.25 reached up to 634.72 and 390.42 mAh·g^−1^; and the discharge capacity was 264.56 mAh·g^−1^ after 100 cycles. In addition, TiO_2_ nanofibers with a TBT/paraffin oil ratio of 2.25 still obtained a high reversible capacity of 204.53 mAh·g^−1^ when the current density returned back to 40 mA·g^-1^. The results confirmed microemulsion electrospinning is indeed a simple and versatile method to prepare porous nanofibers with large specific surface area and the prepared nanofibers improve greatly their electrochemical performance in practical applications.

## Supporting Information

The Supporting Information presents CV curves and Nyquist impedance plots.

File 1 Additional experimental data.
