# Supplementary material for: Fabrication of hierarchically porous TiO2 nanofibers by microemulsion electrospinning and their application as anode material for lithium-ion batteries
Source: Beilstein J Nanotechnol. 2017 Jun 22;8:1297–306. doi: 10.3762/bjnano.8.131 (PMC5496575; doi:10.3762/bjnano.8.131)
Supplement: File 1 — Additional experimental data. [file Beilstein_J_Nanotechnol-08-1297-s001.pdf]

Supporting Information

for

Fabrication of hierarchically porous TiO_2 nanofibers by microemulsion electrospinning and their application as anode material for lithium-ion batteries

Jin Zhang[1,2], Yibing Cai*[1,§], Xuebin Hou[1,2], Xiaofei Song[1,2], Pengfei Lv[1,2], Huimin Zhou[1,2] and Qufu Wei*[1,¶]

Address: [1]Key Laboratory of Eco-textiles, Ministry of Education, Jiangnan University, Wuxi, Jiangsu 214122, People's Republic of China and [2]College of Textile and Clothing, Jiangnan University, Wuxi, Jiangsu 214122, People's Republic of China

Email: Yibing Cai - yibingcai@jiangnan.edu.cn; Qufu Wei - qfwei@jiangnan.edu.cn

* Corresponding author

[§]Tel.: (+86) 510-85912007, Fax: (+86) 510-85912009

[¶]Tel.: (+86) 510-85913653, Fax: (+86) 510-85913100

Additional experimental data

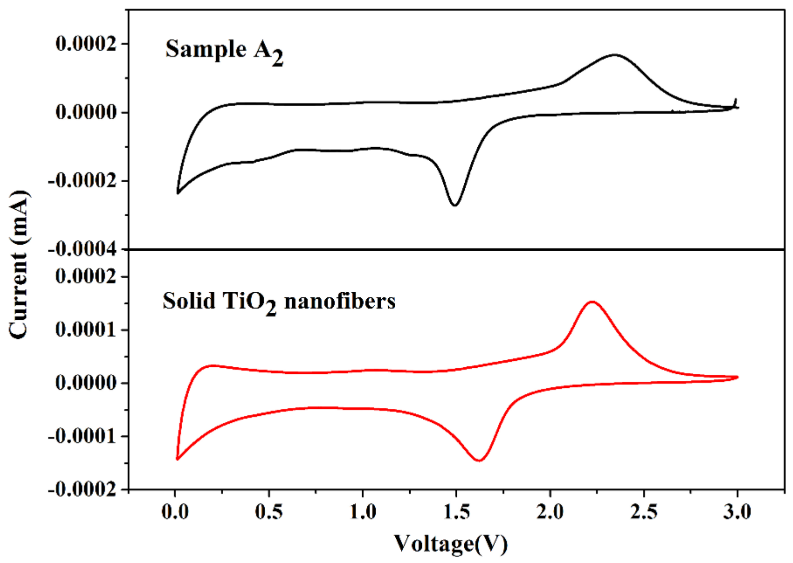

Figure S1: CV curves of Sample A_2 and solid TiO_2 nanofibers.

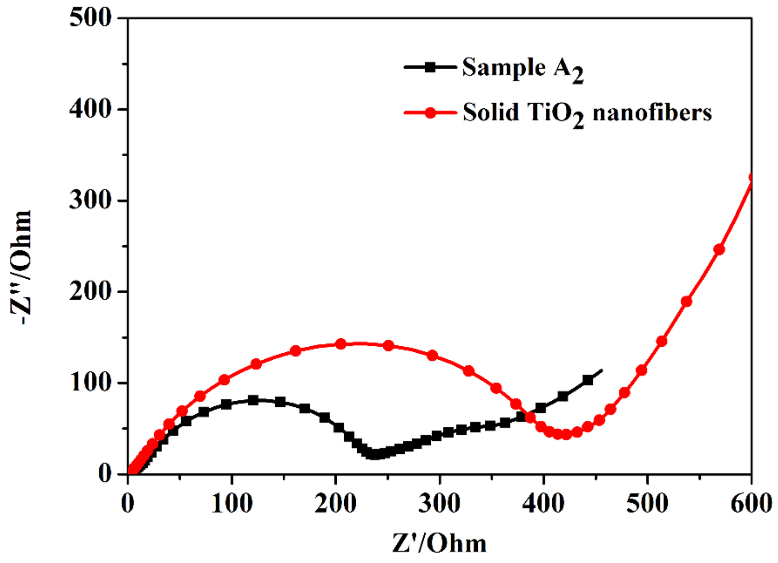

Figure S2: Nyquist impedance plots of TiO_2 nanofiber electrodes made of Sample A_2 and solid TiO_2 nanofibers in a fresh cell.
